# Comparison of sodium chloride hopper cubes grown under microgravity and terrestrial conditions

**DOI:** 10.1038/s41526-019-0085-0

**Published:** 2019-11-11

**Authors:** Donald Pettit, Pietro Fontana

**Affiliations:** 1 NASA Johnson Space Center, Houston, TX 77058 USA; 2 Josef Reinhart-Weg 1, CH 4500 Solothurn, Switzerland

**Keywords:** Fluid dynamics, Solid-state chemistry

## Abstract

Sodium chloride (NaCl) grown in terrestrial conditions form hopper cubes under diffusion controlled mass transport (Péclet number: ≪ 1), high supersaturations (*S* > 1.45), and fast growth rates (10–110 µm/s) over periods only maintainable for seconds to minutes yielding hopper cubes typically <250 µm. Here we report on NaCl hopper cubes grown in microgravity on the International Space Station (ISS) by evaporation of brine. They grew under diffusion limited mass transport (Péclet number: ~4 × 10^−4^ − 4) at low supersaturation (*S* < 1.002) and slow growth rates (0.34–1 µm/min) over periods of days to weeks. Due to the lack of sedimentation, symmetrical hopper cubes, 2–8 mm were produced. The most striking differences between microgravity and terrestrial gravity hopper growth conditions are low supersaturation and slow growth rates over long periods of time. Large, 1–20 cm naturally occurring symmetrical NaCl hopper cubes are found suspended in brine soaked mud, hypothesized to be produced in a slow growth, diffusion dominated environment. We speculate these geologic conditions allow for hopper growth similar to that of microgravity.

## Introduction

The habit of a crystal is given by its internal structure and the external growth conditions. The later are mainly determined by impurities, temperature, supersaturation, mass transfer, heat transfer and sedimentation. During the crystallization process, small differences in concentration and temperature are generated and under the influence of gravity these result in buoyancy driven convection that in turn affects the mass and heat transfer. Once nucleated, symmetrical growing crystals quickly become influenced by sedimentation, typically sinking to the crystallizer bottom. Due to asymmetrical growth conditions, crystallographic identical faces grow with different rates resulting in different crystal habits (e.g. crystals change from cubic to tabular habitus). To maintain symmetrical growth conditions on earth, crystals have to remain suspended in the solution by stirring or freely hanging in the solution or being submersed in a viscous gelatinous medium. In microgravity, density driven convection and sedimentation are essentially eliminated.

There have been a number of prior investigations of aqueous phase crystallization in microgravity. In the 1980s it was found that several protein crystals grown under microgravity were larger with fewer crystalline defects than earth grown crystals. To determine their 3-D structure larger more perfect protein crystals were desired.^1^ Good reviews of protein crystallization and structural analysis are given in.^2,3^

There have been microgravity investigations with aqueous crystallization of inorganic compounds. The synthesis of Zeolites (microporous, aluminosilicates) under microgravity showed that the microgravity crystals were 10–50% larger with significantly smoother crystal surfaces.^4^ Calcium phosphate has been grown at high supersaturation where the size of microgravity grown spherulitic crystals were at least 1.5 orders of magnitude larger than ones grown on earth.^5^

We chose the NaCl crystal system as a case study for aqueous crystallization in microgravity in part because it is well characterized with extensive scientific literature and because of the ease of conducting such nontoxic crystallization experiments on the ISS.

Salt (NaCl) crystals grow in supersaturated brine, where the concentration of NaCl is higher than its equilibrium concentration. They grow by incorporation of Na^+^ and Cl^−^ ions in the cubic crystal structure. The ions are transported by diffusion and convection from the bulk of the supersaturated brine to the crystal surface before they can be incorporated into the crystal structure.

If ionic surface integration of Na^+^ and Cl^−^ is slower than bulk ion transport, the crystal growth is integration controlled (typically under convective dominated conditions). In this case a uniform boundary layer around the growing crystal forms and under symmetrical growth conditions, the layer by layer growth leads to flat faced cubes. These are typical growth conditions at low supersaturations where edges and corners grow with the same rate as the centers of a crystal face.

**Fig. 1:**
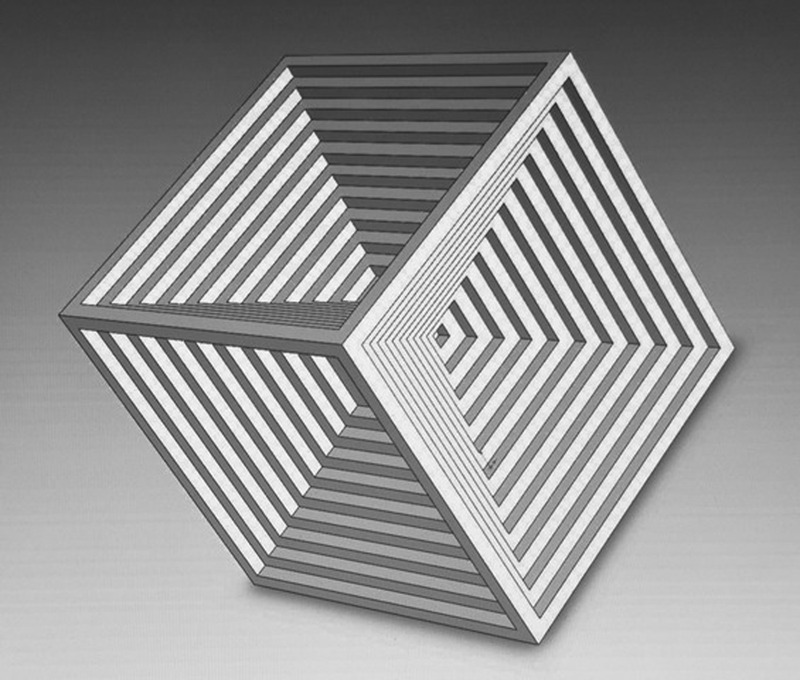
Model of a hopper cube

In diffusion controlled crystal growth the solution surrounding the growing crystal becomes depleted of Na^+^ and Cl^−^ ions. The isoconcentration or isosupersaturation curves are not uniformly distributed around the growing crystal (“Berg effect”). Interferometric measurements reveal that the concentration of ions is highest at corners and edges.^6^ Therefore growth preferentially begins as two dimensional nucleation forming laterally moving steps. When corner and edges grow faster than the face centers, stepped hopper morphology results (hopper growth). Under symmetrical growth conditions hopper cubes form (Fig. 1). The growth of stepped cube faces occurs in the ${\langle 100 \rangle}$ direction.

The specific surface area [m^2^/kg] of hopper cubes and therefore the specific surface energy [J/m^2^ kg] are substantially greater compared with flat cubes. Hopper cubes with their greater surface energies are growth dominated and not equilibrium shapes.

Hopper growth is enhanced with increasing crystal size^7^ and with increasing supersaturation.^8^ Hopper growth is suppressed by forced convection equalizing the concentration differences between corners, edges, and face centers.

Non-uniformity in the growth environment, typically wall effects caused by sedimentation or attachment to brine/air interface, leads to asymmetrical face growth changing the crystal morphology from cube to tabular (thin square plates), either as flat faced or hopper depending on the growth regime.

We present the growth conditions of our microgravity grown NaCl hopper cubes and compare them with earth grown NaCl hopper cubes including naturally occurring hopper cubes grown through geologic processes in brine soaked mud.

## Results

During NaCl crystallization on the International Space Station (ISS) the steady state component of residual acceleration was 1.2 µg (1.2 × 10^−6^ g) causing slow back and forth or circular motions of suspended crystals with approximately a 90-min period (one orbit). We used two crystallizer geometries, a hemispherical 2–3 cm sessile drop and a 0.8-cm spherical drop. For the 2–3 cm diameter crystallizers, time lapse imagery showed that relative motion between bulk suspended crystal faces and the immediate surrounding brine was of the order of 100 µm/min. For the 0.8 cm diameter crystallizers, the bulk brine motion was effectively suppressed giving a relative motion between the hopper cube faces and the surrounding brine too small to be determined from time lapse imagery. The only measurable motion between the crystal faces and the brine in the 0.8-cm diameter crystallizers was due to the actual crystal face growth measured from time lapse video.

The growth rate, G, determined from time lapse video for both crystallizer geometries gave an average value of 0.68 µm/min (STD deviation 0.23 µm/min, extreme spread 0.34–1.0 µm/min). From this growth rate (G), we estimated the supersaturation [*S* = actual concentration/equilibrium saturation concentration] from equation 1 in ref. ^9^ *G* = K(*S*−1)^a^, to be *S* < 1.002 using a growth constant value *K* = 9.0 µm/s corrected for 19 °C from Fig. 4 in ref. ^9^ and their exponent “a” value of 1 for brines with *S* < 1.45.

The Péclet number, Pe, is a dimensionless number important in processes involving convection and diffusion. It is defined as the ratio of convective mass transport to diffusive mass transport and is given by Pe = *lv*/*D* where *l* is the system characteristic length (taken here as the edge length of a hopper cube), *v* is the fluid velocity (taken here as the brine velocity relative to the hopper cube face), and *D* is the diffusion coefficient of Na^+^ Cl^−^ in the brine. The Péclet number defines the degree of diffusion domination (Pe < 1) or convective domination (Pe > 1).

We estimated the Péclet number in the 2–3 cm diameter crystallizers in microgravity using 100 µm/min brine velocity relative to the suspended hopper cube faces, cube edge lengths from 50 µm (smallest measurable nuclei) to 4 mm (the largest edge length we could make video measurements on) and *D* of 1.57 × 10^−5^ cm^2^/s (5.32 mol NaCl/l, saturated conditions at 25 °C.^10^) We calculated at the onset of nucleation (hopper cube edge *l*~50 µm) the Péclet number was ~5 × 10^−2^ and at the largest measurable size hopper cube (*l* ~ 4 mm) was ~4.

For the 0.8 cm diameter crystallizers, the advance of the hopper face due to growth was the only measurable relative brine velocity. Using 0.68 µm/min as the relative velocity between the brine and the hopper cube faces gives a Péclet number at nucleation (*l* ~ 50 µm) of ~4 × 10^−4^ and at final (*l* ~ 4 mm) ~3 × 10^−2^.

Under our microgravity conditions for either crystallizer geometry, initial hopper growth was clearly diffusion limited but at final stages could be dominated by either diffusion or convection.

Table 1 contains all our crystallization data’s obtained under microgravity and compare them with NaCl hopper cube growth regimes under terrestrial laboratory conditions.

**Table 1:** NaCl hopper cubes in microgravity and in normal gravity

	ISS (microgravity)	Terrestrial laboratory (normal gravity) ^9,15,21^
Diffusion	Diffusion dominated at the mm edge length scale in centimeter scale bulk brine crystallizers	Diffusion dominated at the 100 μm edge length scale in sub-millimetre scale bulk brine crystallizers
Supersaturation	low: *S* < 1.002	high: *S* > 1.45
Crystal morphology	hopper cubes, tabular hopper crystals	Crystal morphology changed to hopper growth at supersaturation ratios *S* > 1.45
Growth rate	slow: 0.0057–0.0167 µm/s (0.34–1 µm/min)	fast: 10–110 μm/s Maximum growth rate for flat faced cubes: 6.5 ± 1.8 µm/s
Growth period	days to weeks (periodically replenishing the brine)	seconds to minutes
Peclet number	~5 × 10^−2^ − 4 in 2–3 cm diameter crystallizers ~4 × 10^−4^ − 3 × 10^−2^ in 0.8 cm diameter crystallizers	~10^−3^ − 10^−2^ in 250 μm capillary crystallizers
Cube size	2–8 mm	0.05–0.27 mm

The most striking differences between normal gravity and microgravity are that hopper cubes under microgravity grow at low supersaturation, small growth rates and long periods of time and not under high supersaturation, high growth rate regimes and short periods of time.

Due to the low supersaturation needed for the growth of hopper cubes under microgravity, it is possible to continue hopper growth for days to weeks by periodically replenishing the brine resulting in larger cube sizes.

We speculate that the giant naturally occurring hopper cubes (5–20 cm) found in brine saturated mud (Details are given under Methods) grew in a diffusion dominated environment (Pe ≪ 1) essentially free from sedimentation due to the supporting mud matrix promoting symmetrical face growth over long periods of time where exceptionally large crystal sizes could form. In effect, these terrestrial muds offer an environment for NaCl growth strikingly similar to that in microgravity.

## Discussion

Hopper growth is the result of a kinetic effect. The interplay of diffusion, the degree of supersaturations and the kinetics of step growth are the factors that determine flat faced cubic or hopper morphology. It occurs only in the diffusion controlled growth regime. Hopper morphology forms when the mass transport to the center of the crystal is slower and the edges continue to grow faster. Due to the “Berg effect”^6^ the supersaturation is highest at corners and edges, where two-dimensional nucleation occurs. In nature hopper growth of cube faces is enhanced when the face growth is retarded by interactions with mud particles^11^ or with adsorbed impurities from the solution.^12^

Hopper cubes grow in antisolvent crystallizations under high initial supersaturation. Under these conditions the nucleation rate and the crystal growth rates are very high causing the supersaturation to become quickly depleted resulting in growth times typically of seconds to minutes. Therefore the resulting hopper cubes are small. The median crystal size decreases with increasing supersaturation because the nucleation rate is exponentially and the growth rate is linear dependent on the supersaturation.^13^ In microcapillaries fast growth rates of 10–110 µm/s were measured.^9^

In contrast hopper morphology produced in microgravity is at low supersaturation and low growth rates, conditions maintainable over periods of days. It would take about a week to grow an 8 mm hopper cube requiring daily brine replenishment. As the hopper cubes grew to 3–8 mm, they would be harvested. With brine replenishment, a single sessile drop crystallizer might produce 3–6 hopper cubes a week for about a month until failure to replenish the brine would result in a dried cemented mass.

In NaCl brines there is a metastable limit (a supersaturation below which spontaneously no new nuclei can form ^14^) of *S* ~ 1.03.^15^ Our growing conditions under microgravity with *S* < 1.002 are well within the metastable zone. The lack of new nuclei and thus reduced competition for supersaturation among a large population of small crystals allows for the long term growth of the few initial crystals resulting in large hopper cubes. This is consistent with the general understanding of growth models.

Conditions favorable for hopper growth in terrestrial laboratories are created by a number of methods including microcapillaries, antisolvents, and gels (see Methods section). In microcapillaries, hopper morphology occurs under diffusion limited growth (Pe ~ 10^−3^ − 10^−2^) in the presence of high supersaturation (*S* > 1.45) at length scales of 100–270 µm. Convection is effectively eliminated by the physical size of the crystallizer and surprisingly, results in smaller Péclet numbers than in multi-centimeter sized crystallizers under microgravity where residual accelerations produce slow cyclic motion without sedimentation. With antisolvents, hopper growth occurs at very high initial supersaturation (*S* ~ 2.6) at length scales of 10–270 µm under significant bulk convection (stirring). In microcapillaries and antisolvents, growth rates are fast (10–110 µm/s) with conditions for growth only made for periods of seconds to minutes. In the case of silicagels the supersaturation is generated with HCl. Hopper crystals form under diffusion dominated regimes within a narrow range of high concentrated HCl (10–11.3 mol/l). No information about the resulting degree of supersaturation and the shape and dimensions of hopper crystals was given. No hopper cubes were produced by fast cooling of saturated brine. This is due to the low temperature dependence of NaCl solubility not creating the needed supersaturation.^16^

In microgravity, the absence of convection gave diffusion limited mass transport for nucleation and initial growth (Pe ~ 5 × 10^−2^) at evaporation-limited supersaturation (*S* < 1.002) in crystallizers (2–3 cm) where brine volumes were much greater than the sum of the volumes of the grown crystals. When hopper cubes approached 4 mm, Péclet numbers approached 4 thus resulting in a transition to a convection dominated regime. Since this was nearing final crystal size, hopper morphology remained with some signs of laminar overgrowth. For the 0.8 cm microgravity crystallizers, Péclet numbers remained less than 10^−2^ thus all growth was in the diffusion limited regime. Slow growth over periods of days to weeks was possible. In spite of the slow cyclic microgravity driven motion, sedimentation was effectively eliminated thus cubes remained suspended giving symmetrical faces. Crystals nucleated at or near a surface formed tabular hopper crystals growing into a loosely interconnected crust.

Table 2 summarizes the general growth conditions for the preferred growth of corners and edges between normal gravity and microgravity for three types of NaCl hopper crystals: hopper cubes, tabular hoppers and pyramidal hoppers. Gourmets know the highly prized hopper pyramids under the name “fleur de sel” (expression in French for **“**flower of salt”).

**Table 2:** NaCl hopper morphology in normal gravity and in microgravity

Terrestrial (normal gravity)	ISS (microgravity)
Hopper cubes	Hopper cubes
Hopper cubes form in symmetrical growing conditions. They grow randomly orientated in stirred solutions in the bulk of the brine or displacively in soft mud sediments.	Hopper cubes form in symmetrical growing conditions. They grow randomly orientated in the bulk of the brine.
Tabular hopper crystals	Tabular hopper crystals
Grow as thin square plates attached to the air/brine surface. Upon reaching a size of about 1 mm, they detach from the surface and sink to the brine bottom where they continue to grow as a lumped mass, or they nucleate and grow directly lying on the bottom.	Grow as thin square plates attached to the air/brine interface and remain attached by surface tension forces until they agglomerate into an interlocking crust.
Hopper pyramids (fleur de sel)	Hopper pyramids (fleur de sel)
These are hollow four sided regularly stepped pyramids with an open square base attached to the brine surface with the apexes oriented downward. They grow macro stepwise at elevated temperatures of 25–80 °C in the localized supersaturated surface film. When one face remains in contact with the surface, growth is only at the highest-lying edges.^33^ At ~3–4 mm in size, surface tension forces can no longer keep them attached to the brine surface and they sink to the bottom stopping their characteristic growth. At the surface of a shallow brine pool cm sized floating hopper pyramids have been observed.^34,35^	We did not observe hopper pyramids in microgravity. We speculate hopper pyramid morphology requires gravitational force to keep the apex directed into the bulk brine thus allowing the growth of a regular sequence of new macro steps. Our brine temperature was 19 °C where hopper pyramid morphology requires a minimum of 25 °C. Fleur de sel is naturally produced in solar salt ponds at temperatures of ca 30 °C and 40% rel. humidity.^36^ This temperature is needed to create by evaporation of brine the highly supersaturated surface layer within the stepwise growth occurs.

For Fleur de Sel, our brine temperature of 19 °C in space was well below the known required temperature range (25–80 °C) needed for that morphology to form. Therefore it is not surprising that none were observed. In addition it lacks the gravitational force to maintain the pyramid apex pointed into the brine bulk. To determine that absence of hopper pyramids is due to the lack of gravity we suggest repeating the crystallization of NaCl in microgravity at temperatures higher than 40 °C.

**Fig. 2:**
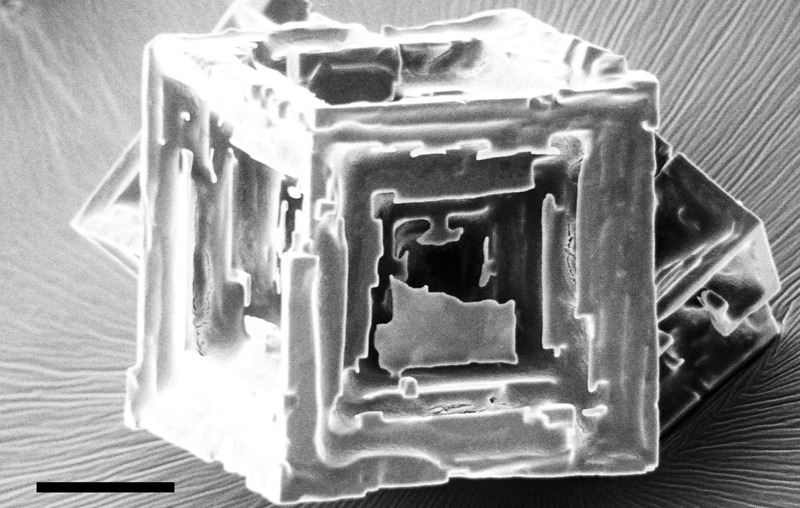
Beginning of a lamellar overgrowth with brine inclusion in an antisolvent grown hopper cube at the end of the crystallization where *S* ~ 1 (Scale bar: 100 µm)^31^

We discuss further the general observation that hopper cubes growth occurs under turbulent (forced convection) conditions. The antisolvent crystallization with MgCl_2_ occurs under stirring-based forced convection. It is well known that stirring reduces the thickness of the diffusion layer and equalizes the concentration gradients around growing crystals both arguments that should prevent the growth of hopper cubes. Kolmogorov^17^ showed that turbulence from forced convection are created with a size distribution ranging from large-scale eddies at the scale of the vessel down to micro-scale eddies dominated by viscosity. Within these micro-scale turbulent eddies, a common engineering assumption is made that viscous-diffusion processes dominate even though they are enveloped within a large-scale turbulent regime. The approximate length scale of these small scale eddies is estimated as *η* ~ *ι*(*vι*/*ν*)^−3/4^ where *η* is the small-scale turbulent length, *v* is the large-scale bulk fluid velocity, *ν* is the kinematic viscosity, and *ι* is the vessel length scale (Equation 1.5.14 from ref. ^17^) The kinematic viscosity is the ratio of fluid viscosity to fluid density *ν* = *μ*/*ρ*, which for saturated NaCl brine at 20 °C, *ρ* and *μ* gives 1197 kg/m^3^ and 1.990 × 10^−3^ Pa-s,^18^ respectively. For our estimate of small scale turbulent size *η*, we used the conditions from our antisolvent experiments (400 ml beaker with internal diameter *ι* of 7.5 cm and a stirring velocity *v* ~ 5 cm/s). This gives an estimate for the length scale of the viscous-diffusion dominated turbulent eddy of ~230 µm. From this we conclude that at least for nucleation and initial growth, the hopper cubes within a micro-scale eddy are under diffusion domination even though submerged within bulk-scale forced convection. As the hopper cubes approach our largest size (270 µm), they may no longer be under diffusion domination, however, this is at the termination of supersaturation and growth where *S* ~ 1 and non-hopper growth (lamellar growth) can be observed on top of the hopper morphology [Fig. 2]. A systematic study of the influence of stirring, turbulent eddy size, and its effect on diffusion limited growth would be worthwhile.

## Methods

We investigated the crystallization of NaCl under microgravity on the International Space Station (ISS).^19,20^ The trace purities in the NaCl brine for these studies are reported in Fontana et al.^19^ The supersaturation was created by evaporation of brine at 19 ± 1 °C, relative humidity of 38 ± 3%, atmospheric pressure of 745–755 mmHg with major constituents 20% oxygen, 79% nitrogen, and 0.5% carbon dioxide.

We used two different geometries of crystallizers; 0.8 cm spherical brine drops affixed to hardened gelatin films in 50 mm diameter stainless steel wire loops (wire diameter 0.6 mm) and sessile hemispherical brine drops fixed to flat polycarbonate substrates with base diameters of 2–3 cm.

**Fig. 3:**
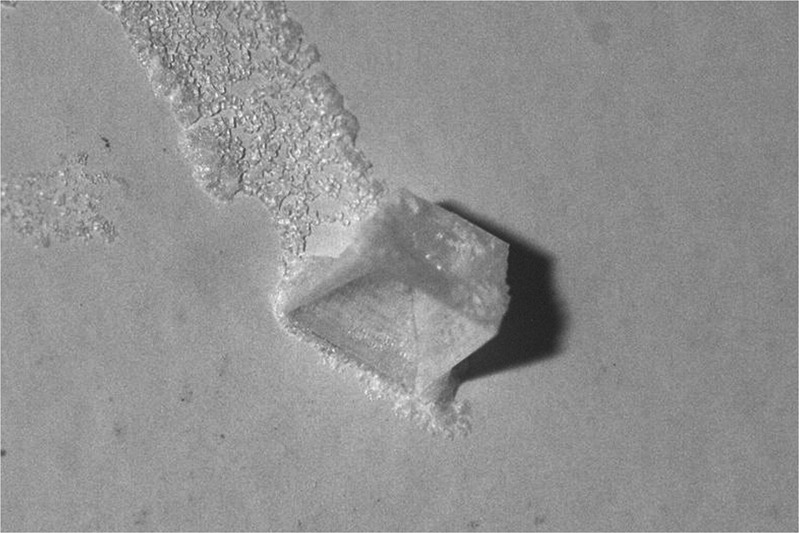
Microgravity grown NaCl hopper cube (edge length: 4 mm)

An example of a 4-mm hopper cube grown within the brine bulk is shown in Fig. 3. This photo was taken shortly after removal from the crystallizer where the hopper cube was attached to a Teflon support by a crust of dried brine.

**Fig. 4:**
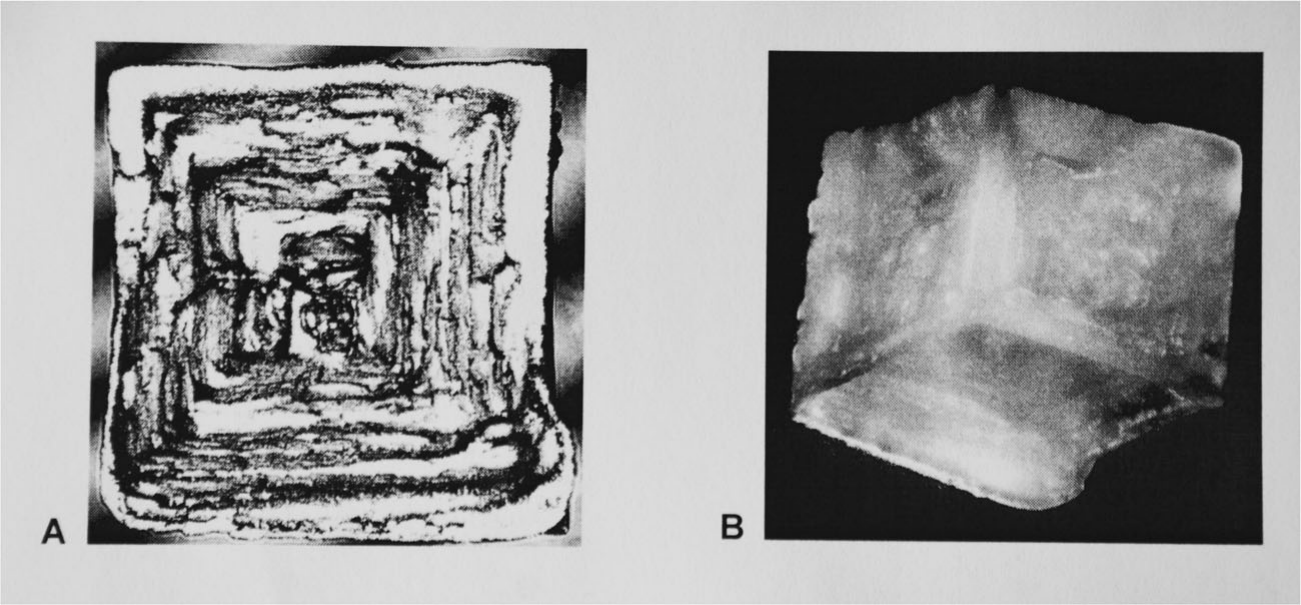
Views of a microgravity grown hopper cube. **a** Depressed, hollow stepped pyramid of a hopper cube standing on a cube face (Laser reflection image). (Edge length: 3.8 mm). **b** The same hopper cube standing on a corner shows three hollow stepped pyramids (Laser transmission image). The crusts are clearly seen in these figures

Another example of a microgravity grown hopper cube with nearly equal sized {100} faces is shown in Fig. 4. When hopper cubes with well-developed inverted step faced geometry were removed from the brine, residual brine adhering to the hopper faces would form a polycrystalline crust within the inverted hopper face. Without the benefit of a desiccator, storage on ISS coupled with the hygroscopic properties of NaCl would result in the polycrystalline crust covering the hopper faces.

**Fig. 5:**
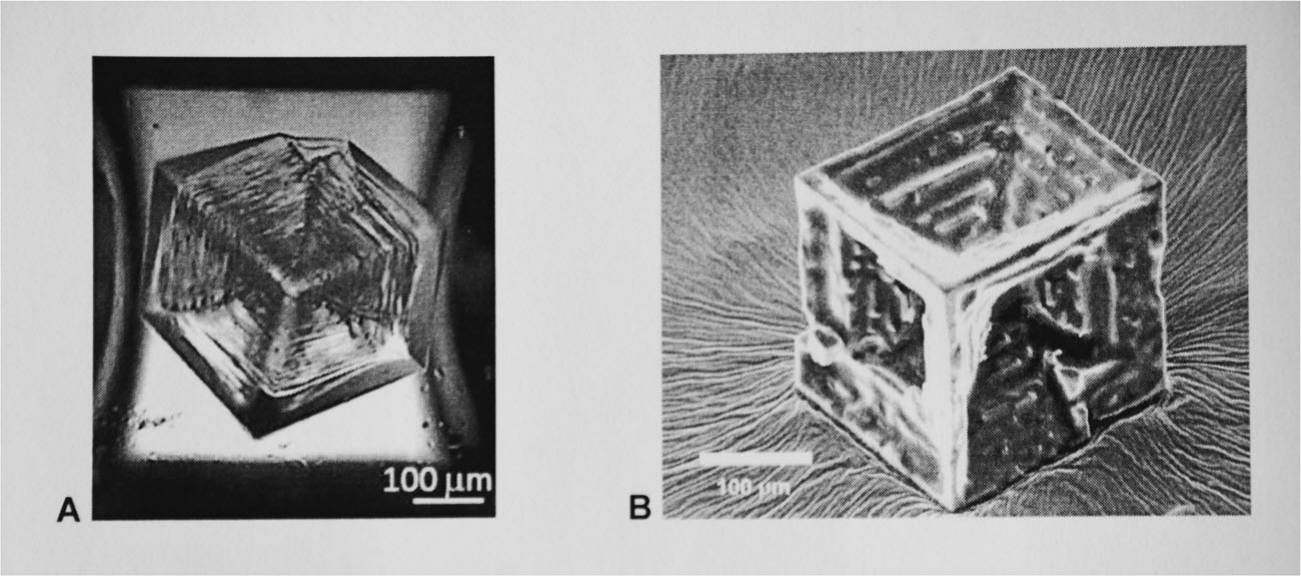
Examples of 2 nearly ideal earth grown hopper cubes. **a** 250 µm NaCl hopper cube grown in a 1 mm capillary: ref. ^15^ Copyright 2014 American Chemical Society. **b** 140 µm NaCl hopper cube with sharp edges obtained with MgCl_2_ antisolvent precipitation]. (Scale bars: 100 µm)

There are several methods for the growth of NaCl hopper cubes under normal gravity. The crystallization of NaCl has been investigated in microcapillaries with diameters of 20–2000 µm^9,15^ by evaporation of brine at 21 °C under a wide range of supersaturation. With these spatially restricted conditions, gravity driven convection could be reduced thus hopper cubes grew in a diffusion dominated regime with Pe ~ 10^−2^ − 10^−3^ at the point of nucleation. Figure 5a shows a hopper cube formed in microcapillaries at *S* = 1.6.^15^

NaCl hopper cubes are obtained by antisolvent crystallization. Magnesium chloride (MgCl_2_) when introduced as an aqueous solution significantly lowers the solubility of NaCl. Precipitation of all available NaCl occurs in about 5 min.^21^ When 1 part of saturated NaCl brine and two parts of saturated MgCl_2_ solution are mixed at room temperature, a high initial supersaturation ratio (*S* ~ 2.6) is produced.^21^ The time scale for mixing is less than that for spontaneous nucleation thus the nucleation and growth of NaCl crystals was in the presence of forced convection. Forced convection kept the NaCl crystals suspended maintaining symmetrical growing conditions. Figure 5b shows a hopper cube we grew through MgCl_2_ antisolvent process.

Gel crystallization is a method to grow NaCl crystals in a sedimentation and convection free environment. Silicagel containing the NaCl solution was supersaturated by the diffusion of hydrochloric acid (HCl) into the silicagel.^22^ The silicagel network within the brine was found not to be integrated into the NaCl crystal structure.^23^ The degree of supersaturation depends on the concentration of HCl. It was observed for HCl concentrations less than 10 mol/l transparent perfect 3 × 3 × 3–4 × 4 × 4 mm NaCl crystals were obtained after 30 days but at concentrations between 10–11.3 mol/l, hopper crystals formed. Above 11.3 mol/l, NaCl dendrites formed.

Naturally occurring NaCl form as halite hopper cubes suspended within mud (silt, clay) and is thus free from normal sedimentation and convection within brine. Euhedral crystal growth is possible because the slowly advancing faces of the growing NaCl crystal push the supporting soft mud out of the way. It is known that at sufficiently low growth rates nearly all small particles (1–15 µm) are pushed ahead by the advancing interface. By increasing the growth rate particles are incorporated in the solid.^24^ Slow growing conditions can produce symmetrical hopper cubes with minimum inclusions of mud.

**Fig. 6:**
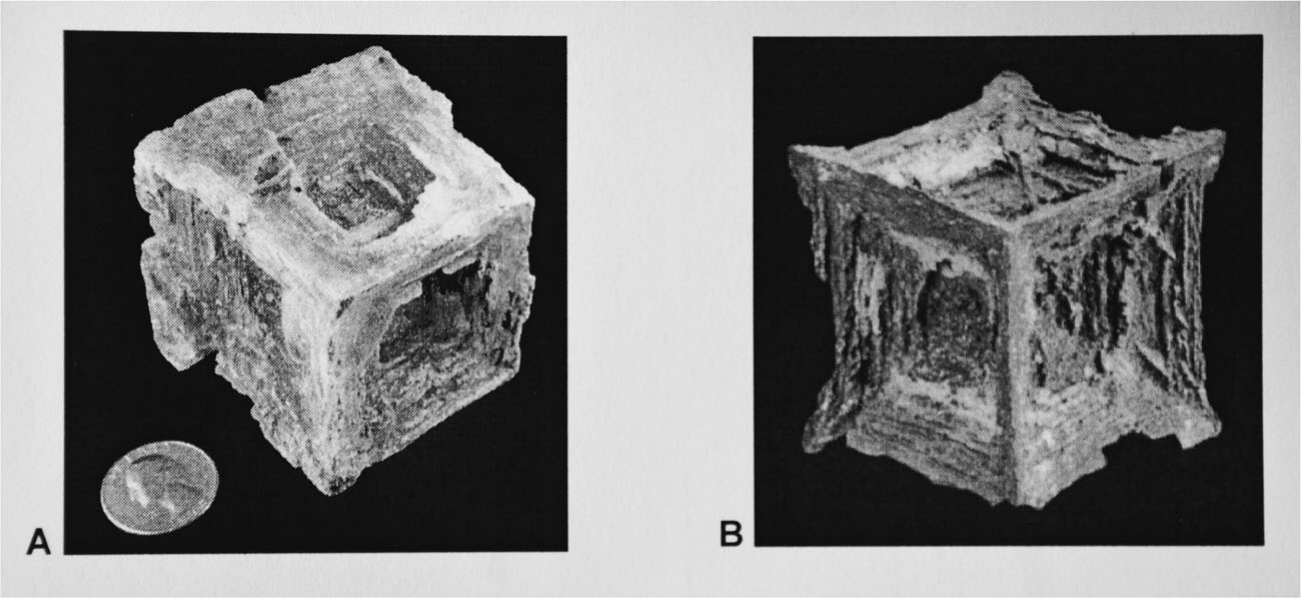
**a** Displacively in mud grown halite hopper and a pseudomorph after halite Displacively grown halite hopper (6.3 × 6.3 × 6.3 cm) with mud inclusions. Reprinted by permission from Springer Nature License: Springer Nature, Encyclopedia of Paleoclimate and Ancient Environments. Mineral Indicators of Past Climates, Gornitz, V. M., Copyright 2008. **b** Hoppershaped calcite pseudomorph after halite covered with reddish-brown Limonite (3 × 3 × 3 cm);^27^ Photo reprinted by permission from: Jesse La Plante (Copyright 2012)^32^

In Dead Sea sediment, 5–10 cm hopper cubes within carbonate mud have been found^11,25^ (Fig. 6a). It is not known if the crystals grew from downward diffusion of Dead Sea brine or from upward diffusion of connate brine.^11^ Mud inclusions have been incorporated into the halite along crystallographic directions during growth, forming a 3D Maltese cross or hourglass formation.^25^ No data exist on the growth rate and the supersaturation of brine during the crystallization nor on the way in which the presence of mud affected the growth rates as in the other examples discussed. Twenty centimeters Hopper cubes were found in Briston Dry Lake, California.^26^ They were suspended in red to green mud and grew displacively in brine soaked clay just below the surface. The halite was clear, nearly inclusion free, with thick clay laminae incorporated along growth planes parallel to the cube faces. It is thought these mud borne halite hopper cubes grew slowly because crystals are both large and clear. It is not surprising that growth without sedimentation can produce large symmetrical crystals.

The morphology of soluble NaCl hopper cubes can also be documented as well-preserved calcite pseudomorph covered with the iron oxide mineral Limonite (Fig. 6b). The pseudomorph was found in the Bolivian highlands near Coro Coro. NaCl grew displacively in a mud matrix and was later replaced by calcite that crystallized in the remaining halite hopper cube voids.^27^

Halite hopper crystals and calcite pseudomorphs after halite are abundant in a mudrock matrix of Alpine rock salt deposits. They formed as displacively grown hopper cubes within mud just during shallow burial. Due to subsequent mud compaction they are often deformed. They have sizes of 2–30 mm. Deformed halite cubes in lithified sediment occur worldwide.^28,29^

Hopper cube pseudomorphs are not restricted to Earth processes. The NASA Mars Exploration Rover Opportunity found by drilling into the sedimentary rock “Lemon Rind“ (North of the Erebus crater) pseudomorphs after a mineral with an apparent cubic crystal habit. Millimeter-scale shapes may suggest the former presence of “hopper crystals” commonly produced by halite in terrestrial evaporates.^30^

### Reporting summary

Further information on research design is available in the Nature Research Reporting Summary linked to this article.

## Supplementary information

Reporting Summary Checklist

## Data Availability

All relevant data are included in the text.

## References

[1] Karr L. J., Miller T. Y., Donovan D. N. NASA: A Researcher’s Guide to: Macromolecular Crystal Growth. https://www.nasa.gov/connect/ebooks/researchers_guide_macromolecular_crystal_growth_detail.html (2017).

[2] McPherson A, DeLucas LJ (2015). Microgravity protein crystallization. npj Microgravity.

[3] Lorber B (2002). The crystallization of biological macromolecules under microgravity: a way to more accurate three-dimensional structures?. Biochimica et. Biophysica Acta.

[4] Coker EN (1998). The synthesis of zeolites under micro-gravity conditions: a review. Microporous Mesoporous Mater..

[5] Lundager Madsen HE (1995). Calcium phosphate crystallization under terrestrial and microgravity conditions. J. Cryst. Growth.

[6] Berg WF (1938). Crystal growth from solutions. Proc. Roy. Soc. Lond..

[7] Nanev Chr (1994). Critical size of crystals growing under diffusion conditions for loss of polyhedral stability. J. Cryst. Growth.

[8] Sunagava, I. *Crystals – Growth, Morphology and Perfection*. Chapter 3 (Cambridge University Press, 2007).

[9] Desarnaud J, Derluyn H, Carmeliet J, Bonn D, Shahidzadeh N (2018). Hopper growth of salt crystals. J. Phys. Chem. Lett..

[10] Chang YC, Myerson AS (1985). The diffusivity of potassium chloride and sodium chloride in concentrated, saturated, and supersaturated aqueous solutions. AlChE J..

[11] Gornitz VM, Schreiber BC (1981). Displacive Halite hoppers from the Dead Sea. J. Sediment. Pet..

[12] Nývlt, J., Ulrich J. *Admixtures In Crystallization*. (VCH, Basel, 1995) ISBN: 3-527-28739-6.

[13] Mersmann, A. *Crystallization Technology Handbook* 718–720 (Marcel Dekker, New York, 2001), ISBN: 0-8247-0528-9.

[14] Dhanaraj, G., Byrappa K., Prasad V., Dudley M. *Handbook of Crystal Growth. Nucleation and Growth Kinetics*, 561–564 (Springer, 2010)

[15] Desarnaud J, Derluyn H, Carmeliet J, Bonn D, Shahidzadeh N (2014). Metastability limit for the nucleation of NaCl crystals in confinement. J. Phys. Chem. Lett..

[16] Langer H, Offermann H (1982). On the solubility of sodium chloride in water. J. Cryst. Growth.

[17] Tennekes, H., Lumley J. L. *A First Course In Turbulence*. 19–22 (MIT Press, 1972)

[18] Lide, D. R. *CRC Handbook of Chemistry and Physics*, 98th Edition, 5–129. (CRC Press, Boca Raton, Florida, 2017/2018).

[19] Fontana P, Schefer J, Pettit D (2011). Characterization of sodium chloride crystals grown in microgravity. J. Cryst. Growth.

[20] Fontana P, Pettit D, Cristoforetti S (2015). Sodium chloride crystallization from thin liquid sheets, thick layers, and sessile drops in microgravity. J. Cryst. Growth.

[21] Raup OB (1970). Brine mixing: an additional mechanism for formation of basin evaporites. Am. Assoc. Pet. Geol. Bull..

[22] Desai CC, Rai JL (1980). Growth of single crystals of NaCl in gels. Krist. und Tech..

[23] Chen L (2014). Gel network incorporation into single-crystals: effects of gel structures and crystal–gel interaction. CrystEngComm.

[24] Kastner M (1970). An inclusion hourglass pattern in synthetic gypsum. Am. Mineralogist.

[25] Gornitz, V. M. Mineral Indicators of Past Climates, *Encyclopedia of Paleoclimate and Ancient Environments*. 573–583 (Springer, 2008)

[26] Handford CR (1982). Sedimentology and evaporite genesis in a Holocene continental‐sabkha playa basin—Bristol Dry Lake, California. Sedimentology.

[27] Brandstetter, R. Corocoro - weltberühmte Pseudomorphosen von Kupfer nach Aragonit, Extra Lapis: Pseudomorphosen **43**, 76–81 (Christian Weise Verlag, München, 2012).

[28] Leitner Ch, Hofmann P, Marschallinger R (2014). 3D-modeling of deformed halite hopper crystals by Object Based Image Analysis. Computers Geosci..

[29] Leitner Ch, Neubauer F, Marschallinger R, Genser J, Bernroider M (2013). Origin of deformed halite hopper crystals, pseudomorphic anhydrite cubes and polyhalite in Alpine evaporates (Austria, Germany). Int. J. Earth Sci. (Geol. Rundsch.).

[30] Squyres SW (2006). Two years at meridiani planum: results from the opportunity rover. Science.

[31] Fontana, P. *Die Vielfalt der Salzkristalle* (P. Fontana, Solothurn, 2013). ISBN 978-3-033-04031-1.

[32] Photo and Copyright (2012): Jesse La Plante P.O. Box 21112 Boulder, CO 80308.

[33] Dellwig LF (1955). Origin of the salina salt of Michigan. J. Sediment. Petrol..

[34] Lowenstein TK, Hardie LA (1985). Criteria for the recognition of salt-pan evaporates. Sedimentology.

[35] Hauschke N, Straub C, Witzke Th (2011). Die Bildung pyramidaler Halit-Hopper in eindunstenden Wasserpfützen. Der. Aufschluss.

[36] Sainz N, Boski T (2019). Is all Fleur de Sel the same? Experience from artisanal saltworks in Castro Marim (Portugal). 10th Symposium on salt (2018): Special issue in Bull. Soc. Sea Water Sci., Jpn..

